# Psychosis Revealing Multiple Sclerosis: A Case Report

**DOI:** 10.1192/j.eurpsy.2026.11094

**Published:** 2026-07-31

**Authors:** R. Chakchouk, S. Frikha, S. Sakka, S. Ellouze, M. Dammak, J. Aloulou

**Affiliations:** 1 Psychiatry B Department, CHU Hedi Chaker Sfax; 2 Neurology Department, CHU Habib Bourguiba Sfax, Sfax, Tunisia

## Abstract

**Introduction:**

The manifestations of multiple sclerosis (MS) are highly variable, mainly including motor, sensory, and cognitive symptoms. However, psychosis as the first manifestation of MS is rare and represents a diagnostic challenge.

**Objectives:**

The aim of this case report is to highlight psychosis as a rare initial manifestation of multiple sclerosis.

**Methods:**

We report a case of a female patient followed in the Neurology Department of Sfax, Tunisia who presented with psychosis as the first symptom of multiple sclerosis.

**Results:**

A 35-year-old female with no prior medical history presented on day 15 postpartum with holocranial headaches and acute psychotic symptoms, including auto and hetero-aggressiveness, visual and auditory hallucinations, delusional ideas, insomnia, and emotional indifference toward her newborn. Physical and neurological examinations revealed no abnormalities and no focal neurological signs. Brain and spinal MRI showed multiple hyperintense lesions in periventricular, juxtacortical, internal temporal, and cervical spinal regions. Isofocalization showed a type 2 profile with an IgG index of 2.17. The remainder of the etiological work-up was negative, supporting the diagnosis of multiple sclerosis. The patient was started on glatiramer acetate and clozapine, which led to a marked improvement of psychiatric symptoms and a sustained remission without subsequent relapses.

**Conclusions:**

This case highlights the importance of considering MS in the differential diagnosis of new-onset psychosis, especially in young adults, to avoid misdiagnosis and ensure timely treatment.

**Disclosure of Interest:**

None Declared

